# Increasing the amplitude of intrinsic theta in the human brain

**DOI:** 10.3934/Neuroscience.2020026

**Published:** 2020-11-05

**Authors:** Pascale Voelker, Ashley N Parker, Phan Luu, Colin Davey, Mary K Rothbart, Michael I Posner

**Affiliations:** 1 Department of Psychology, University of Oregon, Eugene OR, USA; 2 BelCo Eugene OR, USA; 3 Independent contractor, Eugene OR, USA

**Keywords:** anterior cingulate, attention network test, connectivity, primary motor area, theta

## Abstract

In a mouse study we found increased myelination of pathways surrounding the anterior cingulate cortex (ACC) following stimulation near the theta rhythm (4–8 Hz), and evidence that this change in connectivity reduced behavioral anxiety. We cannot use the optogenetic methods with humans that were used in our mouse studies. This paper examines whether it is possible to enhance intrinsic theta amplitudes in humans using less invasive methods. The first experiment compares electrical, auditory and biofeedback as methods for increasing intrinsic theta rhythm amplitudes in the Anterior Cingulate Cortex (ACC). These methods are used alone or in conjunction with a task designed to activate the same area. The results favor using electrical stimulation in conjunction with a task targeting this region. Stimulating the ACC increases intrinsic theta more in this area than in a control area distant from the site of stimulation, suggesting some degree of localization of the stimulation. In Experiment 2, we employed electrical stimulation with the electrodes common to each person, or with electrodes selected from an individual head model. We targeted the ACC or Motor Cortex (PMC). At baseline, intrinsic theta is higher in the ACC than the PMC. In both areas, theta can be increased in amplitude by electrical stimulation plus task. In the PMC, theta levels during stimulation plus task are not significantly higher than during task alone. There is no significant difference between generic and individual electrodes. We discuss steps needed to determine whether we can use the electrical stimulation + task to improve the connectivity of white matter in different brain areas.

## 1. Introduction

White matter change, as measured by Diffusion Tensor Imaging (DTI) in adult humans, has been reported following the performance of cognitive tasks, particularly in studies of working memory and meditation [1], however the extent and generality of these finding remain controversial [2]. In our previous work we found that a month of mindfulness meditation training in comparison with a relaxation training control can increase fractional anisotropy (FA), a measure of white matter efficiency obtained from DTI [3]. It has been frequently reported that meditation increases the amplitude of theta (4–8 Hz) recorded over the frontal midline [4] in electrodes overlying the Anterior Cingulate Cortex [5]. Based on this literature, we hypothesized that a key factor associated with changes in white matter FA following meditation training was the increase in amplitude of the frontal theta rhythm found following meditation [6].

**Figure 1.**
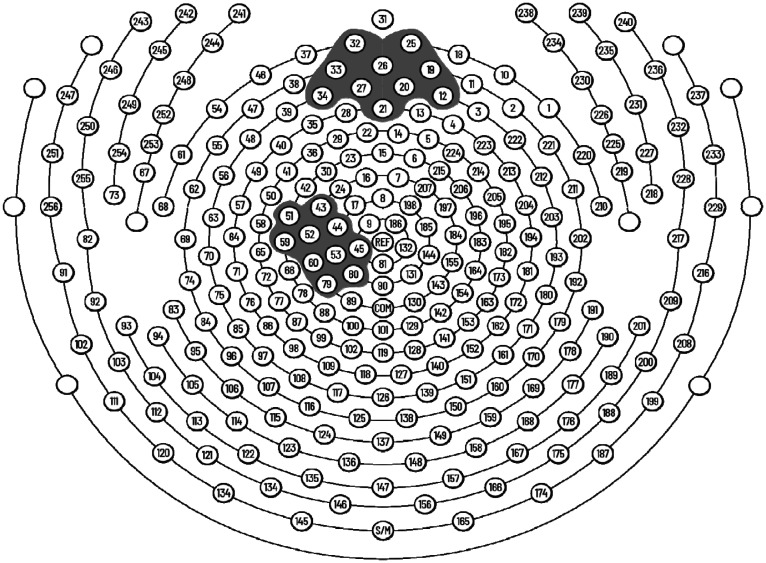
The 256 electrode sites are shown from the Phillips Neuro Geodesic Sensor Net. Electrodes used for stimulation or recording for the ACC (top) and PMC (left) are shown in shaded areas. We used 20 electrodes for each area during stimulation but show only the 10 electrodes used for recording and which lie over the region of interest.

**Figure 2.**
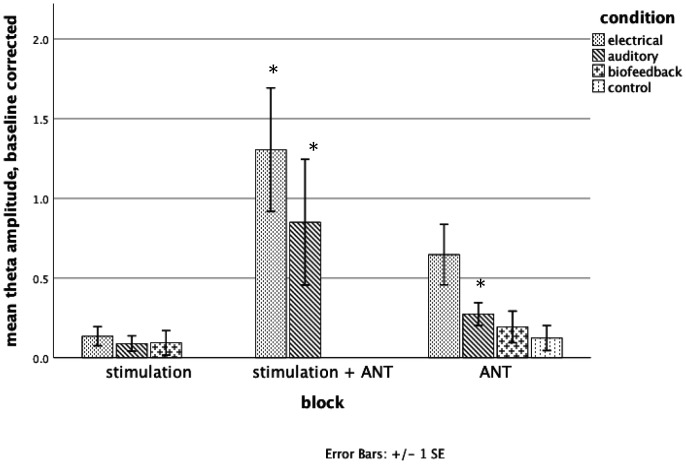
shows theta amplitude above baseline in each condition of Experiment 1. Significant theta above baseline following corrections for multiple tests is shown by asterisk.

To test this idea we developed a mouse model in which implanted lasers produced increased activity within the Anterior Cingulate Cortex (ACC) We found that low frequency stimulation reduced the white matter g ratio (axon diameter divided by axon diameter plus myelin) proximal to the point of stimulation but not remote from it [7]. Moreover, the change in g ratio was correlated with reduced anxiety as measured by reduced time spent in the dark area of a test box [8]. However, the optogenetic methods involved in the mouse study cannot be used with humans. In this paper we seek to compare various less invasive methods of increasing intrinsic theta amplitude that might be applied at all locations in the human brain. These include: (1) electrical stimulation from scalp electrodes (Figure 1), (2) auditory stimulation and (3) biofeedback each performed with and without a task activating the same brain area. A previous study found that combining a task with DC stimulation produced a stronger effect on working memory [9]. If we are able to develop methods that increase intrinsic theta amplitude, we can take the next step of using theta stimulation to determine if we can increase the FA of white matter surrounding the ACC measured by DTI.

Experiment 1 compared the several non-invasive stimulation methods to determine which is most effective for inducing an increase in intrinsic theta rhythms in the general area of the ACC, which is a part of the executive attention system [10]. We compared electrical stimulation, sensory (auditory) stimulation and biofeedback to increase intrinsic theta rhythms. Each was performed either alone or, if possible, accompanied by a task known to activate the ACC.

Based on our mouse study we hypothesized that 6 Hz stimulation from electrodes overlaying the midline frontal scalp region would be the most effective method to enhance intrinsic frontal midline theta activity. We examined the increase in intrinsic theta in electrodes over the frontal midline, thought to reflect ACC activity, with those over an unstimulated control area, the Motor Cortex (PMC). Hypothesis 1 is that electrical stimulation over the frontal midline (Figure 1) will increase intrinsic theta in the ACC more than in the control left Motor Cortex. Hypothesis 2 proposed that performance of the Attention Network Test (ANT) [11] that has been shown to activate the ACC [12], will also increase intrinsic theta. Our third hypothesis was that the combination of electrical stimulation at 6 Hz and the ANT performance would enhance theta amplitude more than either alone.

In Experiment 2, we used the electrical method to compare the effectiveness of the generic electrode configuration used in Experiment 1, which was the same for each participant, with individual electrodes chosen from a head model of each participant. Hypothesis 4 was that the individual configuration will improve intrinsic theta more than the generic configuration, Hypothesis five was the electrical stimulation + task would increase theta amplitude in both the ACC and PMC. Although the motor area has low intrinsic theta activity, we expected intrinsic theta to be enhanced by stimulation when it was the target area. In summary, we hypothesized that the individual electrodes will be more effective for both brain areas and that electrical stimulation during performance of a task that also activates the targeted area will be the best way of increasing theta.

## 2. Experiment 1

### 2.1. Methods

Fifty volunteer undergraduate participants were randomly assigned to one of four groups (20 in electrical; 10 each in auditory, biofeedback and no stimulation). Each participant completed a single session consisting of a fixed sequence of blocks. All procedures were approved by the University of Oregon Institutional Review Board.

All participants were fitted with a 256-channel geodesic sensor net (Philips EGI Neuro, Eugene, OR), and unless otherwise indicated, saline was used to facilitate contact with the scalp. Impedances were kept below 100 kΩ to ensure good contact [13],[14]. Participants were instructed to perform all blocks with their eyes open and to avoid unnecessary movement. The experimental sequence began with measurement of resting state EEG, recorded for two minutes to establish a baseline. This was followed by one minute of stimulation appropriate for their condition which alternated with one minute of no stimulation, for a total of six minutes. The electrical artifact in the stimulation condition swamps the internal theta activity, thus we used the one minute of no stimulation to measure the effect of the prior minute of stimulation.

All participants performed a common behavioral task either alone or in combination with the stimulation. The task chosen was the Attention Network Test (ANT) [11]. This task uses a central arrow pointing left or right as the target. In order to produce conflict, two flanking arrows are used on either side that are either congruent in direction with the central arrow or incongruent. The participants responded to the direction of the central arrow with a left or right key press. This task uses flankers to induce conflict and has been shown to activate the ACC [12]. Participants received instruction for the ANT and were again exposed to alternating one-minute periods of stimulation and no-stimulation while performing the ANT for six minutes. This was followed by a six-minute period of performing the ANT without stimulation. Finally, a two-minute post-test baseline EEG was acquired. Some conditions did not include all blocks (Table 1a). The ANT was used because its target involved either congruent or incongruent flankers. The difference between these two flanker conditions has been shown, in common with many conflict related tasks, to activate the ACC [12].

#### 2.1.1. Stimulation groups

Electrical stimulation. Targeting the ACC with current injected at the scalp requires careful modeling. To target the ACC, we used the 256-channel saline Geodesic Transcranial Electrical Neuromodulation (GTEN) system (Philips Neuro, Eugene, OR, USA). In the present research, we used electrodes chosen from two individual head models in our files that were not part of the present study. In these two individual head models there was a consistent scalp patch for 10 electrodes over the frontal midline. In addition to the common 10 midline frontal electrodes, both had a large distance between the source/sink pairs which demonstrates the requirement for delivering current to deep cortical sources [15],[16], such as the ACC. Therefore, we set ten stimulation electrodes over the frontal midline and the other ten stimulation electrodes over bilateral occipital cortex. This “generic” electrode configuration ensures that the largest amount of current will reach the midline frontal cortex including the ACC, however, other brain areas also received some current. The generic configuration was similar to that found to record ACC activity in a previous study [3]. Current at each electrode pair was set at 100 µA (1 mA across the entire array).

In all electrical conditions alternating current stimulation at a frequency of 6 Hz was used. For electrical stimulation, the electrode contacts of the geodesic sensor net to the scalp were facilitated by a 1:1 saline-Lidocaine paste mixture (4–5% Lidocaine ointment mixed with an equal portion Elefix electrolyte paste) for all 20 injection electrodes and a saline solution (2% w/v KCl) absorbed by sponges for the remaining electrodes. Just preceding data collection, Lidocaine was rapidly absorbed into the scalp using a 1-minute/0.5 mA current DC procedure, this method is intended to mute any tingling or other scalp sensation below the 20 injection electrodes during the electrical condition so that there is less distraction while performing the attention task. The sampling rate of EEG was set at 1000 samples per second for electrical stimulation and 250 samples per second in the other conditions. To examine the amount of theta during a trial we performed a fast Fourier transform at a frequency resolution of 2 Hz.

Auditory stimulation. This method has been used to improve brain theta [17] and has been shown to modify some forms of memory [18]. Our method consisted of the presentation of two tones of 100 and 106 Hz, which produces sound with an amplitude varying at 6 Hz. They were presented over speakers at about 70 db intensity. Pilot studies have shown that this stimulation produces increased theta in the ACC as well as other brain areas.

Biofeedback. This method has been used to induce theta in the human brain [19]. Participants were instructed to develop a relaxed state and were provided a visual display to guide them to produce the highest level of 4–8 Hz frontal activity. Participants were provided with two forms of feedback, their current theta amplitude (as measured second-to-second) and their highest theta amplitude score in that block. Participants in this condition were given a 10-minute practice period to improve their performance before their activity was recorded. The suggested behavioral adjustments were to: (1) maintain slow and steady breathing; (2) relax muscle tension; (3) avoid becoming engaged in thoughts; (4) stay in the present moment; and (5) avoid judgment of thoughts or feelings experienced.

Control. This group was fitted with the net but received no external stimulation. In Eperiment 2 we also used electrodes related to the primary motor cortex (PMC, Figure 1). To deal with the localization of our electrical target we compare the effect of stimulating the ACC from scalp electrodes on the intrinisc theta rhythm in the ACC itself with its effect on theta from PMC. In Table 2.

Experiment 2 stimulation was limited to electrical only. In addition to targeting the ACC, we also used electrodes related to the primary motor cortex (PMC, Figure 1). To deal with the localization of our electrical target we compare the effect of stimulating the ACC from scalp electrodes on the intrinsic theta rhythm in the ACC itself with its effect on theta from PMC in Table 2.

#### 2.1.2. EEG analysis

Stimulus presentation was accomplished with E-Prime (Psychology Software Tools, Sharpsburg, PA, USA), and stimulus-related event flags were automatically relayed to the Netstation software (Philips Neuro, Eugene, OR, US) during the experiment. For passive blocks, flags were generated at one-second intervals.

The continuous EEG data was processed first with a 0.1–50 Hz bandpass filter and then segmented in 500 ms segments using the flags. Five-hundred ms is the approximate amount of time it takes to complete a trial during the ANT task, from target presentation to response, a time where the ACC should be most engaged in the task. Once segmented, the data were mean (DC) corrected.

For each condition, 20 segments of artifact free data were used for analysis. The choice of 20 good segments per givenlock was based on the balance of having enough artifact-free data for analysis vs the inherent structure of the study blocks; often during the stimulation + ANT condition, we would have only 20–30 good segments due to the limited number of trials during the 3 minutes of ‘off’electrical time and movement-related artifacts.

All segments were individually and manually evaluated for possible artifacts (including high amplitudes outside the range of EEG and sharp transients), and 20 of these segments with minimal bad channels and distributed over the length of the block were chosen for inclusion, providing a total of 10 seconds of EEG data per block. For each chosen segment bad channels were replaced using spherical splines and then referenced to the average of all channels. The data were further analyzed using a fast Fourier transform for each block. Theta values were averages of amplitude over the 500 millisec of the 20 segments chosen for analysis, the 4–6 Hz and 6–8 Hz theta levels were then averaged to give a single value.

Figure 1 shows the 256-electrode array, the electrodes are displayed on a horizontal plane and electrode 31 lies between the eyebrows, near the nasion. Within the midline shaded area are the electrodes related to the ACC and within the lateral left shaded area over the left hemisphere are electrodes related to the PMC. We used the PMC as the control to demonstrate that stimulation of the ACC had a greater effect on those electrodes than on the remote PMC. The midline frontal electrodes shown in Figure 1 were used for both stimulation and analysis.

In the electrical stimulation group, any intrinsic frontal EEG is swamped during electrical stimulation. Therefore, the three one-minute periods of nonstimulation that immediately followed electrical stimulation were used in the analysis. We expected a brief carry over of neural synchronization, reflected as oscillations in the theta EEG band, to result from the electrical stimulation. In the auditory and biofeedback conditions we used the three periods of during the one-minute during stimulation as the relevant interval.

The mean theta amplitude of the 2-minute interval at the start of the experiment was used as the theta amplitude baseline. For one participant in the auditory condition, the final baseline was lost. Total amplitude of 4–8 Hz rhythms were calculated for the electrical and auditory conditions and included: baselines, stimulation, stimulation + ANT, and ANT only (Table 1). The ANT was not performed during the biofeedback condition because we expected it to be too difficult for participants to both maintain theta activity from feedback and perform the ANT. Therefore, there is no stimulation + ANT block for this condition. The control condition included only the baselines and the ANT. From the control group we obtained a value for theta activity when performing the ANT in the absence of preceding or concurrent stimulation. We compared the theta activity amplitudes generated using external stimulation for the three methods to see if they differed and to determine which one was most effective.

### 2.2. Results experiment 1

We first present the theta activity at baseline and then with each condition of stimulation. In all conditions we used the baseline before any stimulation to subtract from the measured theta. We have used non normalized data to perform t tests. However, by subtracting the pre-task baseline from the data for each person we did reduce differences between people in skull shape, electrode placement etc. Which were present both in the baseline and under task conditions.

Next we present the behavioral effects in terms of reaction time for each method and condition of the experiment and finally, we cover what we learned about the optimal frequency of stimulation.

**Table 1a.** Order of events in experiment 1.

Block	electrical	auditory	biofeedback	control
1	beginning baseline	beginning baseline	beginning baseline	beginning baseline
2	stimulation alone	stimulation alone	stimulation alone	
3	stimulation + task	stimulation + task		
4	task alone	task alone	task alone	task alone
5	ending baseline	ending baseline	ending baseline	ending baseline

**Table 1b.** Order of events in experiment 2.

	task order
Block	task first generic first	no task first generic first	task first individual first	no task first individual first
1	beginning baseline 1	Beginning baseline 1	beginning baseline 1	beginning baseline 1
2	intro to task	no task + generic stimulation	intro to task	no task+ individual stimulation
3	task + generic stimulation	intro to task	task + individual stimulation	intro to task
4	no task+ generic stimulation	task+ generic stimulation	no task + individual stimulation	task + individual stimulation
5	ending baseline 1	ending baseline 1	ending baseline 1	ending baseline 1
6	task	task	task	task
7	beginning baseline 2	beginning baseline 2	beginning baseline 2	beginning baseline 2
8	task + individual stimulation	no task + individual stimulation	task + generic stimulation	no task + generic stimulation
9	no task + individual stimulation	task + individual stimulation	no task + generic stimulation	task + generic stimulation
10	ending baseline 2	ending baseline 2	ending baseline 2	ending baseline 2

Since we used multiple t tests within Experiment 1 and 2 to assess the effect of the various methods on intrinsic theta we use a Bonferroni correction by dividing 0.05 by the five hypotheses mentioned in our introduction to obtain a conservative significance value of 0.01. In Experiment 1 electrical stimulation plus task produced a significant t value as did ANT alone. In Experiment 2 the electrical condition plus task and task alone all produced a significant values for the ACC and for the PMC. The exact value are discussed below and in the results of Experiment 2. It should be pointed out that even if we used a more radical Bonferroni correction, based on a total of 20 t tests, of 0.0025 the overall conclusion of our paper that electrical stimulation + task produces significant increase in intrinsic theta in both ACC and PMC would hold up.

#### 2.2.1. Overall theta differences

There is a great deal of variability in theta-band activity, particularly in the task and stimulation + ANT task conditions. Since preliminary analysis showed electrical + ANT improved theta the most and to bolster statistical power we ran an additional 10 participants only in the electrical condition. The data for the first ten participants and the second ten participants were similar so we combined them.

All four conditions included a pre- and post-baseline, and we conducted a repeated-measures ANOVA of the two baselines across the four conditions. There was no significant difference between pre- and post-baselines [F (1,45) = 3.04, p = 0.088] or between baselines in the four conditions [F (3,45) = 0.36, p = 0.78], although there was a slight tendency for the post-baseline to be higher than the pre-baseline. Because the pre baseline comes before any stimulation method we used that to test whether each of the methods improves intrinsic theta either alone or in conjunction with the ANT.

In order to characterize the localization specificity of theta activity during measurement, we separately averaged the intrinsic theta amplitude values for the electrical condition at both the frontal midline and a remote location (PMC). Table 2 compares the intrinsic theta measured at the beginning baseline and the minute following electrical stimulation of the ACC, with and without a task and during task alone. A paired-sample t-test showed midline frontal theta activity was greater than theta activity recorded over PMC [t (19) = 3.98, p = 0.001] during the pre-baseline interval. Over all conditions ACC stimulation influenced ACC theta significantly more than it influence the more remote PMC [F (1,152) = 17.78, p < 0.001]. These results show a degree of localization of the electrical stimulation effects found in this experiment.

Figure 2 shows the mean theta amplitude recorded from the midline frontal electrodes in the various conditions of Experiment 1. In the electrical stimulation condition stimulation alone had a small effect on theta amplitude which was not significant following correction for multiple comparisons [t (19) = 2.26, p = 0.036]. Electrical stimulation plus ANT had the largest effect which was significant following Bonferronni corrections [t (19) = 3.37, p = 0.003]. Theta activity during ANT alone was also significantly higher than baseline. [t (19) = 3.42, p = 0.003]. The electrical stimulation plus ANT produced more theta activity than the ANT alone, but this difference did not reach significance [t (19) = 1.81, p = 0.086].

**Table 2.** Experiment 1 mean of theta amplitude (µV) at ACC and PMC sites as a function of ACC stimulation for the baseline, electrical stimulation, task alone and combined electrical stimulation + task. The ACC measured data were derived using the same electrodes used for stimulation targeting the ACC. The PMC data are also taken from Exp 1 with ACC stimulation, but theta changes shown were derived from electrodes over the PMC.

Site of stimulation	ACC
site of recording	ACC	PMC
baseline	1.22	1.00
stimulation alone	1.35	1.09
task alone	1.86	1.25
stimulation and task	2.52	1.27

**Table 3.** Experiment 1 overall mean RT and network scores with stimulation on and following stimulation (standard deviation).

stimulation block	stimulation on				stimulation off			
	RT	conflict	alerting	orienting	RT	conflict	alerting	orienting
electrical	471 (46)	78 (31)	40 (53)	14 (48)	471 (62)	81 (52)	40 (46)	33 (41)
auditory	507 (67)	82 (35)	50 (50)	47 (38)	498 (72)	86 (35)	19 (47)	49 (33)

In the auditory condition, stimulus alone did not produce significantly more theta activity than the baseline [t (9) = 1.85, p = 0.097]. Theta activity during auditory stimulation plus ANT was higher than baseline, but this difference was not significant [t (9) = 2.16, p = 0.060].

The biofeedback condition did not appear to produce significantly more theta activity than baseline. Because the feedback was visual, it was not possible to combine it with the ANT. Stimulus alone did not produce more theta activity than [t (9) = 1.21, p = 0.26].

#### 2.2.2. Reaction times

In general, ANT RT performance during electrical stimulation (Table 3) was very similar whether or not the stimulation was on. The one exception was the orienting effect. Most participants experienced phosphenes during electrical stimulation, which consists of a peripheral flashing light. This effect could have competed with the orienting cue. Among those that experienced phosphenes (n = 17) the orienting score during electrical + ANT trended lower during the minute when the alternating current was applied than during the minute without current [t (16) = 2.021, p = 0.06].

#### 2.2.3. Optimal frequency

In our mouse study where we were able to use optogenetics to control ACC activity we found that lower frequency (1 Hz) stimulation was more efficient than higher frequency (8 Hz) stimulation at improving connectivity. To explore the effectiveness of 6 Hz stimulation in humans on actual neural activity, we divided our amplitude data in the electrical stimulation + task condition in 2 Hz steps from 0.1–8 Hz. We observed a significant peak in amplitude within the frequency range 2–4 Hz [repeated-measures ANOVA within-subject effect F (1.2,22.4) = 8.57, p = 0.006].

### 2.3. Discussion

**Figure 3.**
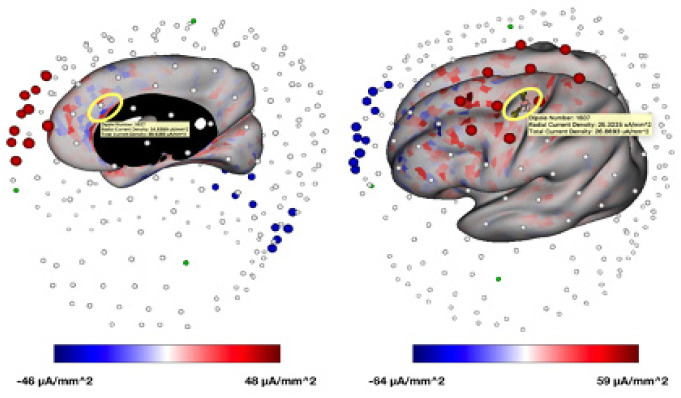
Cortical radial current density (i.e., density of current in direction aligned with direction of pyramidal neurons) model using generic stimulation montages. Left: Cortical current density associated with stimulation montage (anode: red electrodes and cathode: blue electrodes) optimized for ACC target (region highlighted with yellow oval). Right: Cortical current density associated with stimulation montage optimized for PMC target (region highlighted with yellow oval).

Experiment 1 found that electrical stimulation targeting the ACC (Figure 1) together with the ANT produced the greatest increase in intrinsic theta (Figure 2). There is evidence that the electrical effects, while not confined to the ACC (Figure 3) but have a larger effect on intrinsic theta in the targeted region (ACC) than in the control area (PMC). Stimulation alone did not produce a significant increase in theta in any condition. Auditory stimulation together with ANT produced an increase, but this was not significant.

## 3. Experiment 2

In Experiment 1 the electrical condition used the same generic electrodes for stimulation of each person. In Experiment 2 we compare the effect of stimulation using generic electrodes to individual electrodes chosen for each individual by use of a head model constructed from their own structural MRI, as described below [20]. We also used the PMC as a target for stimulation and compared it with the ACC as target. In both cases the corresponding unstimulated area served as a control.

### 3.1. Method

Twenty-four right-handed participants were recruited to participate (18 female), aged 18–50 (Mean 25, SD 9) in this 2-session study. Half were assigned the ACC condition and the other half to the PMC condition. One participant was not able to attend the second session, thus we only collected EEG data from 11 participants in the ACC condition.

A structural MRI scan was taken during the first session, as well as geodesic photogrammetry system [21] images of the participant wearing the geodesic net (Philips Neuro, Eugene, OR). Before the second session the MRI data were processed, using the Modal Image Pipeline (MIP, Philips Neuro, Eugene, OR) to identify six head tissues (scalp, skull, eye balls, cerebral spinal fluid, white matter, and gray matter) and cranial air passage ways. The GPS images were used to reconstruct the electrode scalp positions. The MRI and GPS data were combined to create a head model for each participant. This model was then used to compute current flow through the head volume for each individual. From these electrical head models, the selection of electrode configurations for delivery of current to a desired cortical target can be obtained using the Reciprocity software (Philips Neuro, Eugene, OR), which is based on the reciprocity principle of current flow [13],[15],[16]. Identification of anatomical targets were based on registration of each individual head model to the Talairach atlas and/or structural feature of the region, such as the hand knob for PMC. For each participant 10 ‘source’ and 10 ‘sink’ electrodes were used for targeting each cortical location. For the ACC target, ten electrodes were usually positioned (by the reciprocity principle algorithm) over the region of interest, but this varied slightly between individuals due to differences in underlying anatomy. Because the ACC is a deep structure within the brain, the remaining electrodes were located along the base of the skull and behind the ears, and this also this varied slightly between individuals, in order to direct current flow through the frontal midline and across the ACC [15],[16]. The ten PMC ‘sink’ electrodes tended to be located over the right frontal region, centering around electrode 15 or more lateral, centering around electrode 184. Stimulation parameters for individual stimulation plans was the same as in Experiment 1 (1 mA tACS).

The generic configuration targeting the ACC and stimulation parameters was the same as in Experiment 1. We did not use stimulation of the PMC in Experiment 1 so the generic PMC configuration was selected from among several individuals to overlay the left precentral gyrus, where the hand knob is located. It is expected that there will be some small electrode overlap between individualized and generic electrode (which is meant to target an “average” location) configurations for both ACC and PMC targets, due to the fact that they are generally located in common locations across individuals (e.g., frontal midline for ACC and contralateral for PMC). Indeed, there was an average overlap of 1.2/20 electrodes for the ACC and 4/20 for the PMC.

Within each brain area condition (ACC and PMC), half of the subjects had the generic configuration for the first half of the study followed by the individual configuration and half had the reverse. We also counterbalanced whether the participants had the task plus stimulation first or second. Thus, participants were evenly distributed into 4 groups: task and generic configuration first, no task and generic configuration first, task and individual configuration first, and no task and individual configuration first (Table 1b).

The second session was scheduled approximately 1 week after the first session. The experimental procedure was as follows for the task and generic configuration first participants (as an example): two-minute EEG baseline, introduction to task and short practice, six minutes of electrical stimulation (alternating one minute on/off) in the generic configuration while performing the task throughout the block, six minutes of electrical stimulation only in the generic configuration (alternating one minute on/off), two minutes baseline, four minutes task only, two minutes baseline, six minutes of electrical stimulation (alternating one minute on/off) in the individual configuration while performing the task throughout the block, six minutes electrical stimulation only in the individual configuration (alternating one minute on/off), two minutes baseline. In the ‘no task first’ group the order of the six-minute blocks was reversed in each half of the experiment. In the ‘individual configuration first’ group the generic configuration occurred in the second half of the experiment.

The stimulus presentation was as described for Experiment 1 and used tACS at 6 Hz. During the task event tags were generated at the point of stimulus presentation, otherwise tags were relayed every second during passive blocks. Data was processed as described for Experiment 1, where 20 representative segments of 500 msec were averaged for each block to determine the voltage amplitude (µ volts) for the theta band (4–8 Hz). As in Experiment 1, measurement of theta activity was limited to the three ‘off’ periods during the 6-minute block. Theta-band EEG activity was quantified using the ten ‘source’ electrodes of the generic electrode configurations which overlay each region of interest.

The ACC-dependent task was the same as used in Experiment 1. The PMC-dependent task was a serial reaction time task (SRTT) which presents a target in one of 4 locations and requires the participant to identify the location by pressing one of four keys corresponding to the target location [22]. The stimulus location was present until the participant pressed the corresponding key and then the next target was present immediately. The trials were presented randomly, without repeating a location. This task activates the hand region of the primary motor cortex [23], we selected only right-handed participants who used their right hand for the response and we always stimulated the left PMC.

Behavioral data was also analyzed in order to look at any differences in reaction time associated with stimulation. For the ANT, reaction time and network scores (alerting, orienting, and conflict) were calculated from all correct trials and the error rate was determined. We compared performance during each stimulation block to the task only scores.

To determine differences in the distribution of theta at the two locations (ACC) and motor cortex (PMC), we averaged the change in theta activity in the minute following stimulation during the task + stimulation block at each location over all subjects relative to that of the preceding baseline.

### 3.2. Results

#### 3.2.1. Location of theta activity changes

Table 4 presents data from Experiment 2. In general the frontal midline electrodes show more intrinsic theta activity than electrodes over PMC, even at baseline [paired t-test, t (22) = 3.96, p = 0.001], and show a larger effect of stimulation than does the PMC. An independent samples t-test comparing task + stimulation, measurements at frontal midline electrodes were significantly higher after ACC stimulation than PMC stimulation [t (21) = 6.79, p < 0.001]; whereas, measurements at PMC electrodes were not significantly higher after PMC stimulation than ACC stimulation [t (21) = 0.13, p = 0.90]. However, paired t-tests show that after ACC-targeted stimulation theta activity was marginally higher over frontal midline electrodes than those over PMC [t (10) = 4.04, p = 0.002], and after stimulation targeting the PMC, theta activity levels were higher in electrodes over PMC than those over ACC [t (11) = 2.25, p = 0.046]. Thus, the effects are larger when the target stimulation area and recording area electrodes are the same (e.g. both ACC or both PCC) than when they differ (target ACC and record PMC or target PMC and record ACC), confirming the localization of the stimulation effect found in Experiment 1 (Table 2).

**Table 4.** Mean average theta amplitude in the theta range as a function of where stimulated and where recorded for all conditions of Experiment 2.

site of stimulation	ACC		PMC
site of recording	ACC	PMC	ACC	PMC
baseline	1.21	0.82	1.11	1.02
stimulation alone	1.40	0.77	1.14	1.05
task alone	1.72	1.00	1.20	1.32
stimulation and task	1.70	1.09	1.21	1.29

#### 3.2.2. Baseline

Baseline intrinsic theta was computed four times during the experiment; before and after stimulation targeted to the ACC or PMC. The pre stimulation baseline is significantly higher in electrodes over ACC than those over PMC [paired t-test, t (22) = 3.96, p = 0.001]. In comparing baselines at the beginning (pre-) and end (post-) of the experiment, the ACC and PMC baselines show a significant increase over the course of the experiment [repeated measures ANOVA within-subjects main effect F (1,21) = 8.48, p = 0.008].

**Figure 4.**
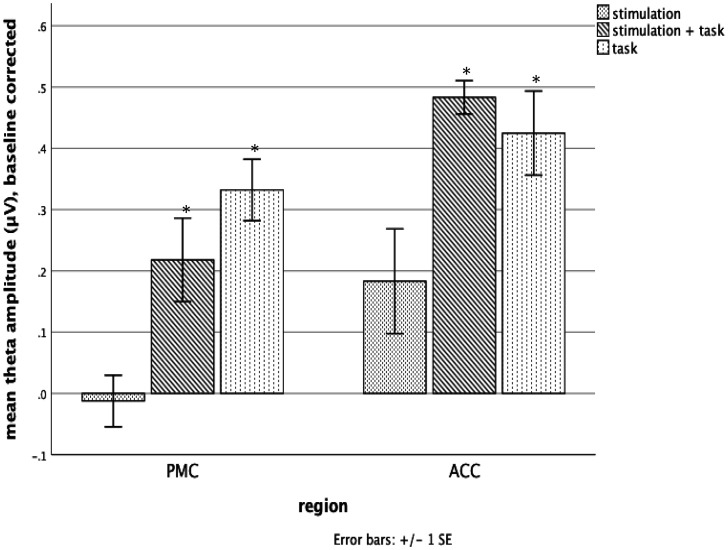
Compares intrinsic theta amplitude above the preceding baseline found in each brain area for the generic electrode configuration. The figure includes stimulation alone, task alone and stimulation plus task which are discussed in detail below. An asterisk indicates the value is significant p ≤ 0.01.

Figure 4 compares intrinsic theta amplitude above the preceding baseline found in each brain area for the generic electrode configuration. The figure includes stimulation alone, task alone and stimulation plus task which are discussed in detail below. The asterisk indicates the value is significant following Bonferroni corrections.

The left panel of Figure 4 indicates intrinsic theta activity measured over the PMC and averaged over 10 participants during stimulation of the PMC, the SSRT task, or both. The right panel indicates intrinsic theta measured over the ACC and averaged over 10 participants following stimulation of the ACC, the ANT task, or both. The baseline preceding stimulation and/or task has been subtracted from each value and only stimulation using the generic electrode configuration is shown.

#### 3.2.3. Stimulation alone

For the ACC, stimulation alone showed an increase in theta activity but it was not significant [t (10) = 2.15, p = 0.057]. The PMC showed no evidence of increased theta activity following stimulation [t (11) = 0.45, p = 0.66].

#### 3.2.4. Task only

The ANT task was performed during ACC blocks while the SSRT task was performed during PMC blocks. Performing the ANT increased intrinsic theta activity measured over the ACC significantly [t (10) = 8.35, p < 0.001]. PMC theta activity was also increased significantly by performing the SSRT [t (11) = 6.64, p < 0.001].

#### 3.2.5. Stimulation plus task

Performing the ANT with electrical stimulation using generic electrodes to target the ACC produced the greatest increase in intrinsic theta activity measured over the ACC relative to baseline, this difference was significant [paired t-test, t (10) = 17.84, p < 0.001]. Performing the SRTT during stimulation of the PMC also provided a significant increase in intrinsic theta activity measured over the PMC relative to baseline [t (11) = 4.48, p = 0.001].

The ACC results were quite similar to Experiment 1, with stimulation alone showing a small non significant increase and the stimulation + task the largest increase. The PMC showed no increase in theta activity by stimulation alone, and task alone had the most theta activity.

### 3.3. Individual vs generic electrodes

**Figure 5.**
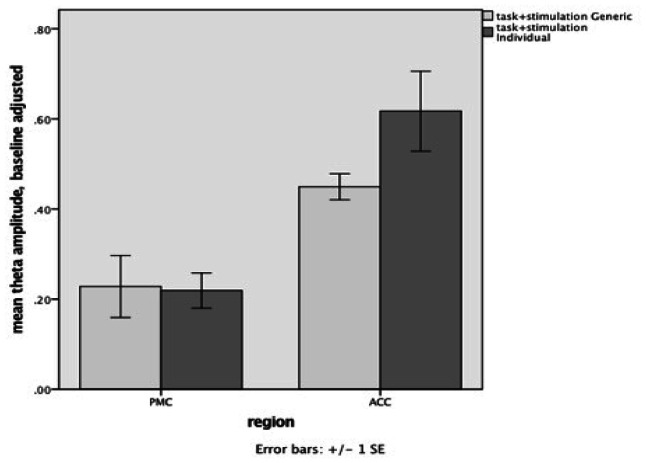
Mean theta amplitude for generic (light) and individual (dark) electrodes in PMC and ACC.

Figure 5 compares individual (dark bar) with generic electrode (light bar) configurations in the stimulation + task condition for both the PMC (left side of figure) and ACC (right side of figure).

Individual electrode stimulation produced a greater increase in intrinsic theta-band activity than did generic electrodes for the ACC but this difference only showed a trend toward significance by t test [t (10) = 1.80, p = 0.10]. For the PMC there was clearly no significant difference [t (11) = 0.32, p = 0.76].

The individual electrode configuration differed from one another and from the generic much more in the ACC than in the PMC. This difference is probably due to the depth of the ACC and the more surface representation of the PMC. In the ACC there is a main effect of generic vs individual in the stimulation plus task condition [repeated measures ANOVA F (1,7) = 5.58, p = 0.05], but much of this is obscured by a large order effect of the stimulation + task to the stimulation only blocks [F (1,7) = 6.80, p = 0.035]. Configuration did not have a significant effect in the PMC.

There is no difference between individual and generic electrodes in PMC and only a small non-significant advantage for the individual electrodes in the ACC.

### 3.4. Behavior

Table 5 shows the mean of the median reaction times for the ACC (ANT task) and the PMC (SSRT task) for two stimulus + task blocks and one task only block. Although RT for the ANT is longer than for the SSRT, there are no significant differences in practice effects between the first and last block of the ANT, while there appears to be a significant practice effect for the SRTT [t (11) = 2.76, p = 0.019]. In both tasks there was no significant effect of stimulation when comparing task only with stimulus + task blocks.

### 3.5. Discussion experiment 2

Overall there was a small non-significant advantage of the individual electrodes for stimulation of the ACC but not for the PMC. The effect of stimulation + task on increasing intrinsic theta was significant in Experiment 2 for both the ACC and PMC. However, for the PMC task alone and stimulus plus task both increased theta about the same amount.

**Table 5.** Reaction times for stimulation + task and task alone as a function of region (ACC vs PMC) and order of stimulation.

		individual first			generic first		
		stim + task block 1	task only	stim + task block 2	stim + task block 1	task only	stim + task block 2
ACC	mean	469.5	449.8	461.4	435.0	465.6	459.0
	SD	60.2	37.7	66.2	21.5	56.6	58.9
PMC	mean	374.9	362.9	361.5	414.7	406.0	404.9
	SD	44.8	36.1	44.2	47.4	52.5	55.9

## 4. Discussion and Conclusions

### 4.1. Findings

Our goal in the current paper was to determine the strongest noninvasive stimulation method to increase intrinsic theta amplitude. Table 4 and Figure 3 show that electrical stimulation from the scalp electrodes, although widespread, does succeed in targeting the intended brain area. The effect of task + electrical stimulation of the ACC was strong for electrodes recording from the ACC but minimal in the PMC. On the other hand, when the PMC was the target, the effect was stronger from electrodes associated with the PMC than those related to the ACC.

Experiment 1 also showed that electrical stimulation plus a task that activated the same area produced the largest amplitude of intrinsic theta. The results for the ACC were similar in Experiment 2. The use of a relevant task to improve the effects of electrical stimulation is in line with some previous findings [9]. We confirmed hypotheses 1, 2 and 3, that 1) electrical stimulation would have a superior effect; 2) the task would enhance intrinsic theta; and 3) electrical stimulation + task condition would produce the strongest overall increase in theta amplitude in the ACC.

The auditory stimulation, although not targeted specifically at the ACC, may have produced an increase when combined with a task, but this was not significant. We did not find any enhancement of the intrinsic theta with biofeedback, although we had a very limited training period, and thus we are unsure whether a better design might produce a different result.

Experiment 2 showed significant improvement in intrinsic theta band activity by task alone or in combination with electrical stimulation in both the ACC and PMC. In the ACC the strongest modification occurred with stimulation + task as found in Experiment 1, but it was not as strong as in Experiment 1. This effect in the ACC was somewhat larger when using individual electrodes but this increase was influenced by task order.

In the PMC, the task alone condition produced as strong or stronger increases in intrinsic theta amplitude than stimulation + task, but both were significantly above their relevant baseline. Stimulation alone was not sufficient to increase intrinsic theta-band amplitudes in the PMC. These results also extend our findings from the ACC to the PMC, a lateral cortical brain area of lower intrinsic theta-band activity.

Our data suggested that the effect of theta stimulation (6 Hz) was somewhat greater in the 2–4 Hz band than in 0–2, 4–6 or 6–8 Hz. This may be related to our previous finding [7] that 1 Hz stimulation in the mouse produced the greatest increase in oligodendrocytes. Thus, while our results favor low frequency stimulation, we must be cautious about concluding which stimulation frequency produces the optimal effect.

Contrary to hypothesis 4 the individual electrodes had only a small and non-significant larger modification of theta amplitude than the generic electrode configuration. Even this small advantage only occurred within the ACC. The advantage of individual electrodes may have been reduced in the ACC by the relatively strong order effects. The individual configuration was more similar to the generic in the PMC. There was also less intrinsic theta measured over the PMC and our conditions had a smaller influence on these theta levels.

Reaction times showed little difference as a result of stimulation condition, but the serial reaction time task was clearly faster than the ANT. There is no strong evidence that the amount of intrinsic theta-band activity found in these conditions lasts longer than the minute following stimulation. However, a small but significant increase in baseline activity found in both brain areas either between the first and second half of the experiment or after stimulation might indicate some small, longer lasting effects and should be followed up.

### 4.2. Conclusions

Our experiment led to the following conclusions: 1. It is possible to increase intrinsic theta by use of scalp electrodes to induce rhythmic electrical stimulation in surface and deep brain areas when accompanied by a task that activates the same area; 2. Current induced by scalp electrodes spreads widely in the brain but does activate targeted areas when appropriate electrodes are chosen; 3. We found some evidence that auditory theta rhythms may increase intrinsic theta in accord with behavioral data of enhanced memory following short term auditory stimulation [17],[18]; 4. Whether a set of generic electrodes used by all participants or individual electrodes chosen by amodel made only modest differences in the obtained results; 5. One negative experience reported by participants undergoing electrical brain stimulation was the occurrence of brief visual phosphenes usually in the periphery and often disappearing after a brief period.

### 4.3. Future studies

Our studies found that electrical stimulation plus a relevant task was most effective in increasing intrinsic theta. We hypothesize that this method of stimulation can produce change in human white matter as measured by FA from DTI, if performed over an extended period, in a manner comparable to the effect of meditation training with humans [24] and laser stimulation with mice [7]. Even with brief theta stimulation there are reports of behavioral changes in memory and attention [19],[25],[26], these may be due to the role of theta in the production of synaptic plasticity due to long term potentiation [27]. If white matter is changed following long-term stimulation, we can also determine if the change in white matter yields faster speeds of responding in tasks involving the ACC and requiring the resolution of conflict. Our study of the PMC showed that in some areas the task alone was sufficient to increase theta activity over task + stimulation. We also hope to determine whether other brain areas can be targeted to induce similar changes in intrinsic activity and, over long term, white matter. If white matter restoration is possible with these means it would be useful to determine if it would aid in recovery of disorders that involve white matter deficits.
